# Elevated Risk for HIV-1 Infection in Adolescents and Young Adults in São Paulo, Brazil

**DOI:** 10.1371/journal.pone.0001423

**Published:** 2008-01-09

**Authors:** Katia Cristina Bassichetto, Denise Pimentel Bergamaschi, Solange Maria Oliveira, Marylei Casteldelli Verri Deienno, Reginaldo Bortolato, Heloíza Vilma de Rezende, Thaís Arthur, Helena Tomiyama, Colyn Watkins, Fabio Mesquita, Maria Cristina Abbate, Esper Georges Kallas

**Affiliations:** 1 Public Health Department of São Paulo, São Paulo, Brazil; 2 Department of Allergy and Immunology, University of São Paulo, São Paulo, Brazil; 3 Henfil Counseling and Testing Center, São Paulo, Brazil; 4 Specialized Attending Service in STD/Aids Campos Elíseos, São Paulo, Brazil; 5 Specialized Attending Service in STD/Aids Lapa, São Paulo, Brazil; 6 Pirituba Counseling and Testing Center, São Paulo, Brazil; 7 Division of Infectious Diseases, Federal University of São Paulo, São Paulo, Brazil; University of California at San Francisco, United States of America

## Abstract

**Background:**

Recent studies have sought to describe HIV infection and transmission characteristics around the world. Identification of early HIV-1 infection is essential to proper surveillance and description of regional transmission trends. In this study we compare people recently infected (RI) with HIV-1, as defined by Serologic Testing Algorithm for Recent HIV Seroconversion (STARHS), to those with chronic infection.

**Methodology/Principal Findings:**

Subjects were identified from 2002–2004 at four testing sites in São Paulo. Of 485 HIV-1-positive subjects, 57 (12%) were defined as RI. Of the participants, 165 (34.0%) were aware of their serostatus at the time of HIV-1 testing. This proportion was statistically larger (p<0.001) among the individuals without recent infection (n = 158, 95.8%) compared to 7 individuals (4.2%) with recently acquired HIV-1 infection. In the univariate analysis, RI was more frequent in <25 and >59 years-old age strata (p<0.001). The majority of study participants were male (78.4%), 25 to 45 years-old (65.8%), white (63.2%), single (61.7%), with family income of four or more times the minimum wage (41.0%), but with an equally distributed educational level. Of those individuals infected with HIV-1, the predominant route of infection was sexual contact (89.4%), with both hetero (47.5%) and homosexual (34.5%) exposure. Regarding sexual activity in these individuals, 43.9% reported possible HIV-1 exposure through a seropositive partner, and 49.4% reported multiple partners, with 47% having 2 to 10 partners and 37.4% 11 or more; 53.4% of infected individuals reported condom use sometimes; 34.2% reported non-injecting, recreational drug use and 23.6% were reactive for syphilis by VDRL. Subjects younger than 25 years of age were most vulnerable according to the multivariate analysis.

**Conclusions/Significance:**

In this study, we evaluated RI individuals and discovered that HIV-1 has been spreading among younger individuals in São Paulo and preventive approaches should, therefore, target this age stratum.

## Introduction

As of June 2006, Brazil was home to over 400,000 confirmed acquired immunodeficiency syndrome (AIDS) cases, with nearly 62% reported in the Southeast region. Seventeen percent of these cases were from São Paulo, the most populous city in the country, and the location of the present study. Our study, then, seeks to better characterize the local and national epidemic through investigation of recent infection in the São Paulo Metropolitan area, the epicenter of the Brazilian HIV/AIDS epidemic.

Despite fairly extensive national surveillance programs, the Brazilian Ministry of Health has recently requested novel methods to generate a more detailed map of the Brazilian epidemic [1]. To that end, the current study utilizes an innovative testing strategy known as Serological Testing Algorithm for Recent HIV Seroconversion (STARHS). Unlike prevalence and case reporting data, STARHS effectively differentiates between RI individuals, defined by infection occurring on average within the previous 170 days, and those with longstanding or chronic infection [2], [3]. This method, therefore, allows us to construct a more explicit map of the leading edge of the epidemic. Such information is critical to the identification of populations currently most in need of prevention efforts. In previous studies, STARHS successfully gathered epidemiological data in other well-stabilized, urban epidemics of San Francisco [4], and New York City [5], in the US, and Santos [6], [7] and São Paulo [8], [9], in Brazil.

While a better understanding of epidemiological dynamics in São Paulo may help alleviate regional HIV-1 transmission, it also has global implications. The diversity of the São Paulo epidemic in terms of age, gender, and mode of transmission may provide insight into other HIV epidemics with shared characteristics. Additionally, the size and diversity of the São Paulo epidemic holds enormous potential for future studies. Identification of these high-risk groups in São Paulo should facilitate enrollment in future studies evaluating a variety of preventative strategies including pre-exposure prophylaxis, post-exposure prophylaxis, candidate HIV vaccines, microbicides, and circumcision. Taken together, a more detailed map of the São Paulo epidemic will promote more focused efforts to slow HIV transmission in a variety of settings as well as aid the development of universally relevant prevention strategies.

The São Paulo public health network provides fertile grounds for the use of approaches that detect recent infections, including the STARHS technique. It consists of an extensive network of basic health care units that include HIV counseling and testing sites (CTS) as well as sexually transmitted diseases (STDs) and HIV/AIDS specialty units (SU) [10]. These specialized network sites afford the unique opportunity to access a large cohort of people who may have been recently infected. Additionally, the nature of these sites allows us to gather detailed information on behavioral, clinical, and social profiles. Finally, the geographical breadth of this urban service network allows us to assess recent infection in a diverse assortment of epidemiological profiles.

## Methods

### Objectives

The present study seeks to better define the epidemiological profiles of individuals presently acquiring HIV-1 infection in Sao Paulo, Brazil.

### Participants

This observational study included users of four service sites of the SMS/SP (two SAE – Campos Elíseos and Lapa; and two CTA – Henfil and Pirituba) who tested HIV-1 positive, from May 2002 to April 2004. During this period approximately 60 individuals were identified as Recent Infection (RI), as defined by the original protocol [11].

### Description of Procedures

#### Serological Testing

Blood was collected in vacuum tubes, allowed to clot, and transported on the same day to the central laboratory of the CSPPHD. Two enzyme immunoassays (EIA) were run. Samples with two non-reacting EIA results were considered negative. Those with indeterminate or reacting results in one or two EIA assays were submitted to a confirmatory Western-Blot assay. If the result was indeterminate or positive, the volunteer was asked to donate a second sample for HIV-1 testing, in accordance with Brazilian Ministry of Health Guidelines [12].

Any sample with a positive result obtained at the central laboratory of the CSPPHD was flagged and an aliquot was transported to the Retrovirology Laboratory at the Federal University of São Paulo. These samples were again analyzed using the STARHS. Briefly, the samples were re-tested using the Vironostika HIV-1 Microelisa System (BioMerieux, Raleigh, NC, USA) EIA, which employs testing of a 1∶20,000 dilution of the specimen under modified incubation conditions. The Vironostika negative control, CDC calibrator, CDC high positive control and CDC low positive control were all diluted and incubated in the same manner as the specimens described above. Samples were initially screened (one well per sample) and all the controls were run in triplicate. The sample optical density (OD) value was standardized as follows: SOD = sample OD−average negative control OD÷average CAL OD−average negative control OD. Samples with screening SOD below 2.0 were re-tested in triplicate in a confirmatory test. The SOD was calculated using the median OD of the triplicates. A sample with SOD below 1.0 in the confirmatory mode was classified as an infection probably acquired in the last 170 days. The result of STARHS was transmitted to the four sites within 10 days after the blood was drawn.

Since the sample could be submitted to a STARHS assay if it were positive at the first round of testing, all the volunteers were informed about the test and were offered the opportunity to sign an informed consent authorizing it, if the standard immunoenzymatic assay resulted positive [11].

#### Volunteer enrollment

At the returning visit of any subject after the first blood was drawn, the test result and counseling were offered at the four testing sites. If the subject tested positive for HIV-1 antibodies, a second blood sample was taken to confirm the first result, again in accordance with Brazilian Ministry of Health Guidelines [12].

A subject identified as recently infected according to the STARHS assay, the volunteer was offered the option to participate in a protocol involving prospective clinical visits and blood collection at the Outpatient Clinic of the Federal University of São Paulo. If the volunteer accepted, a second informed consent was explained and offered, allowing participation in the protocol. A rapid HIV-1 antibody test and a hematocrit were performed using a fingertip puncture. Females were also asked for a urine sample for a dipstick pregnancy test. Volunteers younger than 18 years old, with a negative rapid test, a hematocrit below 28%, or who were pregnant, were not enrolled in the study. The subjects were then submitted to the second blood drawing for further tests, and were asked to come to a scheduled visit at the Federal University of São Paulo within 15 days. CD4+ T lymphocyte counts, and plasma viral load were also determined [11].

#### Data sources

The source documents consisted of routine questionnaires used in the participant sites. The data was transcribed by a formula created specifically for this study, including dates (birth and collection of blood), serological status (HIV-1 and VDRL), results of test for recent infection, knowledge of their serological status at time of collection, participating unit, city and neighborhood of residence, demographics (gender, age, education, occupation, marital status, race, family income), behavior variables (drug use, type of exposure, sexual practices, condom use), and risk variables (partner behavior, number of partners, DST referred).

As an analytical resource, new variables were constructed from selected characteristics (condom use, occupation, and partner risk), combining information from the original questions with those from field “observations”, with the final goal of a more extensive analysis. While “condom use” was initially considered a question that approached use without exception, the field “observations” required the generation of three different categories: *always* (since the initiation of sexual activity); currently always, but not in the past/never and occasionally (since the initiation of sexual activity). For “occupation”, the variable “employment status” was created with three categories: employed (any paying occupation), unemployed/without pay (includes students, inmates, or homemaker), and retired. After information became available for a large part of participants, the variable “non-injection drugs” was created and dichotomized into subcategories user and non-user. For the categories related to the variable “partner risk”, we used a hierarchy of risk described in the consensus of the “Comitê Assessor de Vigilância Epidemiológica do Programa Nacional de DST/AIDS” [13].

Because we utilized data from another source document (and did not generate the information prospectively), we evaluated their quality by creating an informational completeness variable, adapting recommendations given for the evaluation of information systems published by the Centers for Disease Control and Prevention, United States [14]. It reflected the amount of missing information based in the common variables in all of the questionnaires; it is expressed in percentage, varying between 0% and 100%; and subsequently classified in three categories (<50%, 50–69.9%, >70%).

### Ethics

The study was approved by the Institutional Review Boards from the Federal University of Sao Paulo and the Public Health Department of the City of São Paulo, and all the volunteers signed the approved informed consent forms before enrollment.

### Statistical Methods

We considered the dependent variable the type of infection (recent and non-recent), whereas the remaining were considered explanatory or control variables. Due to the statistical significance found between the age and recent infection, we opted to analyze the data considering the age (in years) in three age groups (14–24.9; 25–59.9, 60.0–70.0), with widespread data only for those with evidence of significant statistical association based in p value (recent infection, employment status, marital status, type of exposure, injection drug use). For the association study we used the Pearson Chi-squared test or Fisher's exact test [15]. The multivariate analysis was driven with a logistical regression model beginning with the variables that exhibited p values <0.20 in the univariate analysis and subsequently excluded those that did not contribute to the distinction of RI based on the maximum likelihood-ratio test [16]. Epi Info [17] and Stata [18] programs were used for the construction of the database and data analyses.

## Results

Data was compiled from 485 HIV-1 positive individuals who visited four sites of the SMS/SP (49% - Henfil, 41% - Campos Elíseos, 6% - Lapa e 4% - Pirituba). Of this total, 57 (12%) of the individuals were discovered to be recently infected with HIV-1. The distribution of RI individuals according to service site was different (p = 0.043): 16% (Henfil); 11% (Pirituba); 8% (Campos Elíseos) and 7% (Lapa). Of the participants, 88% resided in the City of São Paulo, 10% within the Greater São Paulo and the remainder (2%), in the State countryside or in another State.

In order to evaluate the quality of our questionnaire data, we used an informational completeness index. An 85% median index of informational completeness was documented (minimum of 23% and maximum of 100%). In 69% of the questionnaires, this index was equal to or greater than 70% and in 4% it was less than 46%. It was observed that independent of the result of the serological test indicating recent HIV-1 infection, the proportions of this index were similar (p = 0.112).

In the univariate analysis, we detected the existence of a statistical association only between recent infection and age, with the greatest portions of recently infected among the participants <25 years-old and those 60 years or older (p<0.001, Tables 1 and 2).

**Table 1:** Participant characteristics and variable distribution in the two groups, with corresponding *p* values.

Characteristic	Recent infection				Total	*p* value(1)
	Yes		No			
	n	%	n	%	n (%)	
Overall	57	11.8	428	88.2	485 (100)	
Gender						
Male	46	12.1	334	87.9	380 (78.4)	
Female	11	10.5	94	89.5	105 (21.6)	0.646
					485 (100)	
Age (years)						
14.0–19.9	4	28.6	10	71.4	14 (2.9)	
20.0–24.9	20	24.4	62	75.6	82 (16.9)	<0.001*
25.0–44.9	31	9.7	288	90.3	319 (65.8)	
45.0–59.9	0	-	55	100.0	55 (11.3)	
60.0–70.9	2	13.3	13	86.7	15 (3.1)	
					485 (100)	
Ethinicity						
Caucasian	18	10.6	152	89.4	170 (63.2)	
African descent	2	6.9	27	93.1	29 (10.8)	0.290
Mulatto	7	10.3	61	89.7	68 (25.3)	
Asian descent	1	50.0	1	50.0	2 (0.7)	
					269 (100)	
Education background						
Illiterate	1	20.0	4	80.0	5 (1.1)	
1^st^ degree incomplete	14	8.2	156	91.8	170 (36.5)	0.171*
1^st^ complete/2^nd^ incomplete	18	14.9	103	85.1	121 (26.0)	
2^nd^ complete/Univ. incomplete	22	14.8	127	85.2	149 (32.0)	
University complete	1	4.8	20	95.2	21 (4.5)	
					466 (100)	
Marital status						
Married	5	7.5	62	92.5	67 (22.3)	
Single	29	15.7	157	84.3	185 (61.7)	0.081
Separated/widow	3	6.3	45	93.8	48 (16.0)	
					300 (100)	
Employment status						
Employed	44	12.9	297	87.1	341 (74.5)	
Unemployed	9	9.0	91	91.0	100 (21.8)	0.454*
Retired	3	17.7	14	82.4	17 (3.7)	
					458 (100)	
Family income (MW = R$200,00)						
<1 MW	4	7.8	47	92.2	51 (23.0)	
1–3	8	10.0	72	90.0	80 (36.0)	0.459
4 or more	13	14.3	78	85.7	91 (41.0)	
					222 (100)	

(1) Pearson Chi-squared test.

* Fisher's exact test.

MW: minimum wage.

**Table 2:** HIV risk behavior among RI and non-RI participants with corresponding *p* values.

Characteristic	Recent infection				Total	*p* value(1)	
	Yes		No				
	n	%	n	%	n (%)		
Route of HIV acquisition							
Sexual	43	12.8	294	87.2	337 (89.4)	0.293	
Blood borne	2	7.1	26	92.9	28 (7.4)		
IVDU	0	-	12	100.0	12 (3.2)		
					377 (100)		
Sexual behavior							
Heterosexual	17	8.6	181	91.4	198 (47.5)	0.198	
Homosexual	21	14.6	123	85.4	144 (34.5)		
Bisexual	10	13.3	65	86.7	75 (18.0)		
					417 (100)		
Condom use							
Always	0	-	3	100.0	3 (0.9)	0.189	
Not in the past/never	13	8.3	144	91.7	157 (45.8)		
Occasionally	26	14.2	157	85.8	183 (53.4)		
Partner risk behavior							
Multiple sexual partners	21	16.7	105	83.3	126 (49.4)		
Blood contamination	3	17.7	14	82.3	17 (6.7)	0.505	
HIV-infected	13	11.6	99	88.4	112 (43.9)		
					255 (100)		
Number of sexual partners							
None	1	12.5	7	87.5	8 (3.3)	0.978	
1	5	16.1	26	83.9	31 (12.6)		
2 to 10	16	13.9	99	86.1	115 (46.7)		
11 or more	12	13.0	80	87.0	92 (37.4)		
					246 (100)		
STD							
Yes	14	8.4	152	91.6	166 (34.2)	0.102	
No	43	13.5	276	86.5	319 (65.8)		
					485 (100)		
VDRL							
Reactive	11	16.9	54	83.1	65 (23.6)		
Non-reactive	28	13.3	182	86.7	210 (76.4)	0.468	
					275 (100)		
IVDU							
Yes	9	8.2	101	91.8	110 (32.2)	0.695	
No	22	9.5	210	90.5	232 (67.8)		
					342 (100)		

(1) Pearson Chi-squared test.

IVDU: intravenous drug use. STD: sexually transmitted diseases

The majority of the participants in the study were male (78.4%), in the 25–45 years-old age group (65.8%), white (63.2%), single (61.7%), with family income of four or more times minimum wage (41%), and distributed uniformly according to degree of education, excluding the illiterate and those with completed university degrees. For behavioral characteristics and risk of HIV-1 infection, we observed that the majority reported *sexual* (89.9%) as exposure type, and the most frequent sexual preference categories were *heterosexual* (47.5%) and *homosexual* (34.5%). A little more than half referred to the use of condoms *sometimes* with a similar distribution in the *multiple partners* (49.4%) and *HIV-1 positive partner* (43.9%) partner risk categories. Nearly 47% described *2–10 sexual partners* and 37.4% exhibited *11 or more*. As far as drug use, 32.2% reported non-injection drug use (alcohol, marijuana, cocaine and crack cocaine). Approximately one-fourth exhibited reactive results for VDRL indicating active or previous syphilis infection and 34.2% reported some STDs in the past (syphilis, genital discharges, warts, gonorrhea, herpes, and hepatitis, Tables 1 and 2).

Of all the participants, 165 (34.0%) reported knowledge of their serological status at the time of HIV-1 testing. This proportion was statistically larger (p<0.001) among the individuals without recent infection (n = 158, 95.8%) compared to 7 individuals (4.2%) with recently acquired HIV-1 infection.

The association study between age and explanatory and control variables indicated that among the youngest participants, the majority were employed (p<0.001), single (p =  0.007), and sexually exposed (p = 0.038). We observed more non-injection drug use (p = 0.046) among participants younger than 60 years of age (Table 3).

**Table 3:** Distribution of individuals according to age stratum, socio-demographic characteristics, behavior, and risk for HIV-1 in four units of the São Paulo Municipal Network of Health, May 2002–April 2004.

Characteristics*	Age (years)			*p* value
	14.0–24.9	25.0–59.9	60.0–70.0	
	(n = 96; 19.8%)	(n = 374; 77.1%)	(n = 15; 3.1%)	
Recent infection (n = 485)				
Yes	24 (25.0)	31 (8.3)	2 (13.3)	<0.001
No	72 (75.0)	343 (91.7)	13 (86.7)	
Total	96 (100)	374 (100)	15 (100)	
Employment status (n = 458)				
Employed	71 (74.7)	265 (75.7)	5 (38.5)	<0.001
Unemployed	23 (24.2)	75 (21.4)	2 (15.4)	
Retired	1 (1.1)	10 (2.9)	6 (46.2)	
Total	96 (100)	350 (100)	13 (100)	
Marital status (n = 300)				
Married	11 (21.2)	55 (22.7)	1 (16.7)	
Single	41 (78.9)	141 (58.3)	3 (50.0)	0.007
Separated/widow	0 (-)	46 (19.0)	2 (33.3)	
	52 (100)	242 (100)	6 (100)	
Route of HIV acquisition (n = 377)				
Sexual	75 (98.7)	251 (87.2)	11 (84.6)	
Blood borne	1 (1.3)	24 (8.7)	2 (15.4)	0.038
IVDU	0 (-)	12 (4.2)	0 (-)	
Total	76 (100)	288 (100)	13 (100)	
IVDU (n = 342)				
Yes	26 (40.0)	84 (31.5)	0 (-)	0.036
No	39 (60.0)	183 (68.5)	10 (100.0)	
Total	65 (100)	267 (100)	10 (100)	

* Unrepresented are data for sex, color, education, sexual preference, partner risk, condom use, number of partners, VDRL and STD due to lack of statistical association.

For the multivariate analysis, we included the following variables: age (years), education, marital status, sexual preference and STD. However, the final model was composed only of the age variable, indicating that the strength of occurrence of RI is greater among younger individuals (*odds ratio* = 0.926; CI 95% 0.881–0.974; p_Wald_ = 0,003).

## Discussion

The present study investigated risk factors associated with recent HIV-1 infection in a cohort of HIV-1 infected patients. The study cohort consisted of primarily sexually exposed individuals, with heterosexual preference the most common sexual identity (47.5%). STARHS testing revealed that the 14–24.9 year-old age stratum suffered the highest proportion of recent infection (25%). The substrata within this group, the 14.0–19.9 and 20–24.9 year old strata, exhibited the highest individual proportions of RI (28.6% and 24.4%, respectively). After multivariate analysis, the 14–24.9 year-old age stratum maintained an independent association with RI (p<0.001). Age, most specifically youth, was the only variable to maintain this significant association with RI after multivariate analysis.

However, despite increased risk for RI among the youngest stratum, younger participants accounted for a minority of the cohort's total HIV-1 infections. We found that the older 25–44.9 year-old stratum accounted for a greater proportion of the total number of HIV-1 infections than the 14–24.9 year old stratum (65.8% and 19.8%, respectively p<0.001). This discrepancy, then, suggests less frequent use of STD/AIDS sites by younger individuals. Taken together, our study indicates that younger individuals in the city of São Paulo are not only at greatest risk for incident HIV-1 infection, but also least likely to seek out STD/AIDS testing and counseling resources.

Despite various studies seeking to characterize risk factors for recent HIV-1 infection, there is no consensus as to which age, if any at all, has a significant association with RI. A study conducted with a primarily MSM cohort in San Francisco, USA, found an association between RI and age greater than or equal to 36 years-old [4]. Conversely, a British study seeking to characterize patients with newly acquired HIV-1 infection reported that younger age was associated with RI [19]. Other studies, one of which was conducted in Santos, Brazil, found no association between RI and age [6], [20]. Our study, therefore, presents new data confirming that, indeed, adolescents and young adults may be the most vulnerable populations.

Despite their increased risk of RI, younger participants accounted for a relatively small proportion of our study cohort's total HIV-1 infections. Brazilian public HIV surveillance confirms this apparent inconsistency. In 2006, municipal surveillance data reported that the >20–39 year old age stratum accounted for the majority of new cases of HIV infection in São Paulo. In contrast, 13–19 year-olds accounted for a relatively small portion of new cases [21]. This discrepancy between RI risk and number of reported infections suggests that HIV case reporting is not capturing the leading edge of the epidemic. Instead, such HIV reporting may suffer from a sample bias as a consequence of fewer young attendees at STD/AIDS service sites. It must be noted, however, that a lower proportion of youths in the City's age distribution cannot explain poor attendance as expansive population pyramids best describe urban Brazilian demographics [22]. Consequently, it seems that despite their greater risk for recent HIV acquisition, younger individuals may be least likely to seek out HIV/AIDS resources.

Previous studies suggest that younger individuals have poor risk awareness and may be less likely to use HIV/AIDS resources. A study conducted in the United States with a MSM cohort reported a significant association between younger age and decreased HIV testing rates [23]. In this study, denial of HIV risk factor was the main reason for testing avoidance. An additional study conducted in southern Brazil reported that among pregnant women, those younger than 18 years of age were least likely to have been tested for HIV [24]. Neglect of HIV/AIDS testing and counseling resources among young people must be addressed. Our study indicates that young individuals are most at risk for RI, and it is therefore important HIV-1 infections in the youth population are both diagnosed and reported. This may prove very useful in proper therapeutic intervention as well as better characterization of epidemiological transmission dynamics.

The increased vulnerability to RI among younger individuals may be attributable to a number of factors. However, increased sexual activity in this age group and the recent success of HAART may most significantly promote risk for RI. A study consisting of interviews throughout Brazil observed that the 15–24 year old age group reported earliest sexual debut and highest percentage of multiplicity of partners [25]. Further, a study conducted in the city of São Paulo with a young MSM cohort reported an association between optimistic perceptions about AIDS and unprotected sexual practices [26]. Other studies have documented similar associations between increased risk behavior and HAART availability [27], [28]. Certainly, the increasing availability of antiretroviral drugs across Brazil may promote decreased risk perception across all age groups. Unfortunately, the greater frequency of sexual contact and partner availability among younger individuals may compound the consequences of such perceptions in this age stratum.

It is worth noting that univariate analysis reported an association between the most elderly and RI. While it was lost after multivariate analysis, this trend remains intriguing because multiple partnership, sildenafil use, and lower rates of condom use have all been previously reported in elderly individuals [29], [30]. Future studies seeking to confirm or refute this trend are warranted.

### Limitations

Our study has some limitations. By using sentinel STD/AIDS service sites, we may have introduced a sample bias. Many users are from high-risk populations and may be more frequent attendees of these clinics. It is therefore possible that users of these sites will have inflated proportions of RI due to increased testing frequency rather than actual greater risk of HIV-1 acquisition. Another limitation is related to the STARHS approach. STARHS may give false negative results in patients with advanced AIDS, those receiving HAART, and elite controllers [2]. However, our inclusion of prospective individual clinical evaluations of participants allowed us to determine whether a patient was currently receiving HAART. This, therefore, mitigates false negative detection of RI related to advanced AIDS and HAART. Although elite controllers still present the possibility of false negatives, they likely make up only a small portion of the cohort. Further, the expected equal distribution of elite controllers across age strata would have little effect on our RI results.

Despite these limitations, our study provides important information on the current state of the epidemic in the City of São Paulo. Our observation of increasing risk for RI and a contrastingly small proportion of total number of HIV-1 infections in the youngest age strata indicates that HIV case reporting cannot accurately describe current epidemiological trends. Additional studies further refining the STARHS technique will be useful in characterizing the latest trends in the HIV/AIDS epidemic. Perhaps more importantly, this study helps define epidemiological trends in the City of São Paulo, the nation's most populous city and the epicenter of the Brazilian epidemic. We observed that younger individuals are not only most at risk for recent HIV-1 acquisition, but are also least likely to seek out testing services. Consequently, additional studies investigating risk behavior and motives for poor attendance among youths at STD/AIDS clinics are warranted. Finally, our study recommends future prevention efforts concentrated towards younger individuals and creative methods seeking to increase youth attendance at STD/AIDS service sites.

## References

[1] Brasil. Ministério da Saúde. Secretaria de Políticas de Saúde. Coordenação Nacional de DST e Aids (2002). Vigilância do HIV no Brasil: novas diretrizes.

[2] Janssen RS, Satten GA, Stramer SL, Rawal BD, O'Brien TR, Weiblen BJ (1998 Jul 1). New testing strategy to detect early HIV-1 infection for use in incidence estimates and for clinical and prevention purposes.. Jama.

[3] Rawal BD, Degula A, Lebedeva L, Janssen RS, Hecht FM, Sheppard HW (2003 Jul 1). Development of a new less-sensitive enzyme immunoassay for detection of early HIV-1 infection.. J Acquir Immune Defic Syndr.

[4] Schwarcz SK, Kellogg TA, McFarland W, Louie B, Klausner J, Withum DG (2002 Oct 1). Characterization of sexually transmitted disease clinic patients with recent human immunodeficiency virus infection.. J Infect Dis.

[5] Des Jarlais DC, Perlis T, Arasteh K, Torian LV, Beatrice S, Milliken J (2005 Aug). HIV incidence among injection drug users in New York City, 1990 to 2002: use of serologic test algorithm to assess expansion of HIV prevention services.. Am J Public Health.

[6] Alves K, Shafer KP, Caseiro M, Rutherford G, Falcao ME, Sucupira MC (2003 Apr 15). Risk factors for incident HIV infection among anonymous HIV testing site clients in Santos, Brazil: 1996–1999.. J Acquir Immune Defic Syndr.

[7] Sucupira MC, Caseiro MM, Alves K, Tescarollo G, Janini LM, Sabino EC (2007 Feb). High levels of primary antiretroviral resistance genotypic mutations and B/F recombinants in Santos, Brazil.. AIDS Patient Care STDS.

[8] de Freitas Oliveira CA, Ueda M, Yamashiro R, Rodrigues R, Sheppard HW, de Macedo Brigido LF (2005 Mar). Rate and incidence estimates of recent human immunodeficiency virus type 1 infections among pregnant women in Sao Paulo, Brazil, from 1991 to 2002.. J Clin Microbiol.

[9] Diaz RS, Kallas EG, Castelo A, Rawal BD, Busch MP (1999 Jul 30). Use of a new ‘less-sensitive enzyme immunoassay’ testing strategy to identify recently infected persons in a Brazilian prison: estimation of incidence and epidemiological tracing.. Aids.

[10] Brasil. Ministério da Saúde. Secretaria de Políticas de Saúde. Coordenação Nacional de DST e Aids (2002). Sistema de informação dos Centros de Testagem e Aconselhamento em Aids SI-CTA: manual de utilização.

[11] Kallas EG, Bassichetto KC, Oliveira SM, Goldenberg I, Bortoloto R, Moreno DM (2004 Dec). Establishment of the serologic testing algorithm for recent human immunodeficiency virus (HIV) seroconversion (STARHS) strategy in the city of Sao Paulo, Brazil.. Braz J Infect Dis.

[12] Brasil. Ministério da Saúde (2003). Portaria 59 de 31/01/2003.

[13] Brasil. Ministerio da Saude. (2002). Programa Nacional de DST e Aids.. Boletim Epidemiológico - AIDS.

[14] Centers for Disease Control and Prevention (2001). Updated guidelines for evaluating public health surveillance systems: recommendations from the guidelines working group.. MMWR.

[15] Snedecor GW, Cochran WG (1967). Statistical Methods. Sixth ed.

[16] Hosmer-Jr DW, Lemeshow S (2001). Applied Logistic Regression. 2nd ed.

[17] Dean AG, Arner TG, Sunki GG, Friedman RI (2002). Epi Info, a database and statistics program for public health professsionals.

[18] StataCorp (2001). Stata Statistical Software. release 7.0 ed.

[19] Chawla A, Murphy G, Donnelly C, Booth CL, Johnson M, Parry JV (2007 Feb). Human immunodeficiency virus (HIV) antibody avidity testing to identify recent infection in newly diagnosed HIV type 1 (HIV-1)-seropositive persons infected with diverse HIV-1 subtypes.. J Clin Microbiol.

[20] Puchhammer-Stockl E, Schmied B, Rieger A, Sarcletti M, Geit M, Zangerle R (2005 Jan). Low proportion of recent human immunodeficiency virus (HIV) infections among newly diagnosed cases of HIV infection as shown by the presence of HIV-specific antibodies of low avidity.. J Clin Microbiol.

[21] Secretaria Municipal da Saúde - São Paulo (2007). Boletim Epidemiológico de Aids/DST e Hepatites B e C do Município de São Paulo..

[22] IBGE - Instituto Brasileiro de Geografia e Estatística. http://www.ibge.gov.br.

[23] Kellerman SE, Lehman JS, Lansky A, Stevens MR, Hecht FM, Bindman AB (2002 Oct 1). HIV testing within at-risk populations in the United States and the reasons for seeking or avoiding HIV testing.. J Acquir Immune Defic Syndr.

[24] Rosa H, Goldani MZ, Scanlon T, da Silva AA, Giugliani EJ, Agranonik M (2006 Apr). Barriers for HIV testing during pregnancy in Southern Brazil.. Rev Saude Publica.

[25] Szwarcwald CL, Barbosa-Junior A, Pascom AR, de Souza-Junior PR (2005 Oct). Knowledge, practices and behaviours related to HIV transmission among the Brazilian population in the 15–54 years age group, 2004.. Aids.

[26] da Silva CG, Goncalves Dde A, Pacca JC, Merchan-Hamann E, Hearst N (2005 Oct). Optimistic perception of HIV/AIDS, unprotected sex and implications for prevention among men who have sex with men, Sao Paulo, Brazil.. Aids.

[27] Ostrow DE, Fox KJ, Chmiel JS, Silvestre A, Visscher BR, Vanable PA (2002 Mar 29). Attitudes towards highly active antiretroviral therapy are associated with sexual risk taking among HIV-infected and uninfected homosexual men.. Aids.

[28] Stolte IG, Dukers NH, Geskus RB, Coutinho RA, de Wit JB (2004 Jan 23). Homosexual men change to risky sex when perceiving less threat of HIV/AIDS since availability of highly active antiretroviral therapy: a longitudinal study.. Aids.

[29] Cooperman NA, Arnsten JH, Klein RS (2007 Aug). Current sexual activity and risky sexual behavior in older men with or at risk for HIV infection.. AIDS Educ Prev.

[30] Stall R, Catania J (1994 Jan 10). AIDS risk behaviors among late middle-aged and elderly Americans. The National AIDS Behavioral Surveys.. Arch Intern Med.

